# Comparison and selection of light-emitting diodes (LEDs) for disease assays on plant pathogenic viruses and bacteria in greenhouses

**DOI:** 10.1371/journal.pone.0328277

**Published:** 2025-08-11

**Authors:** Anne K. J. Giesbers, Barendinus J. A. van Doorn, Joanieke van Oorspronk, Carla Oplaat, Christel de Krom, Maria Bergsma-Vlami, Annelien Roenhorst

**Affiliations:** Netherlands Institute for Vectors, Invasive plants and Plant health (NIVIP), National Plant Protection Organization (NPPO), Netherlands Food and Consumer Product Safety Authority (NVWA), Wageningen, The Netherlands; National Chung Cheng University, Taiwan & Australian Center for Sustainable Development Research and Innovation (ACSDRI), AUSTRALIA

## Abstract

Supplemental lighting in greenhouses has traditionally been dominated by high-pressure sodium (HPS) lights. However, a shift towards light-emitting diode (LED) technology is gaining momentum due to its energy efficiency, customizable light spectra, and adjustable light intensity, which together allow for more precise control over plant development. In plant pathogen diagnostics, where symptom expression is essential, HPS lights have typically been used in greenhouse settings. Since LEDs are generally optimized to promote plant growth and quality, they may affect plant physiology, including responses to pathogens. To investigate how different lighting sources affect the expression of viral and bacterial disease symptoms, two types of LEDs with different spectra were compared with the traditionally used HPS lights. LEDs with a “daylight” spectrum, featuring pronounced blue and red peaks resulted in poor virus symptom expression, though the expression of bacterial symptoms was less affected. In contrast, LEDs with a broad spectrum – characterized by a modest blue peak, a prominent red peak, and a small far-red peak – elicited virus and bacterial symptoms similar to those observed under HPS lights, when adjusted at equal light intensity level. This study provides insights into symptom development in plants inoculated with viruses and bacteria under various lighting conditions, highlighting the influence of light intensity and spectrum. Based on the results of this comparative study, “broad spectrum with far-red” LEDs were identified that are suitable for disease assays on plant pathogenic viruses and bacteria.

## Introduction

Light-emitting diodes (LEDs) are increasingly adopted as an energy-efficient alternative to high-pressure sodium (HPS) lights for supplemental illumination in greenhouses. Whereas HPS lights generate high levels of radiant heat, LEDs are far more efficient at converting electricity into photosynthetically active radiation, thereby reducing energy usage and associated costs [1,2]. However, since LEDs generate less heat, additional energy is often needed to maintain optimal greenhouse temperatures for plant growth, particularly in colder periods. As a result, in comparison to HPS lights additional supplementary heating is required, especially during winter. However, it is generally more efficient to manage heating and lighting separately, allowing for precise control over each factor to support plant growth and reduce overall energy consumption [3]. The total energy savings achieved by a transition from HPS to LED technology in greenhouses has been estimated to be in the range of 10–25%, accounting for both lighting and heating needs [2]. Consequently, adopting LED technology can play a significant role in advancing the global energy transition and enhancing the sustainability of greenhouse horticulture.

Another advantage of LEDs is their relatively long lifespan. Modern horticultural LEDs are designed to maintain at least 90% of their initial light output for an average lifespan of 45 000 hours [4]. In comparison, commonly used HPS lights, such as the MASTER SON-T PIA Plus, achieve the same performance for only 16 000 hours [5]. Beyond longevity, LEDs offer customizable light spectra and intensity, enabling optimization of plant development, quality and metabolism [6]. In contrast, HPS lights have a limited spectrum and minimal adaptability. Moreover, as the horticultural industry increasingly shifts from HPS to LED technology, the availability of HPS lights is declining and is prompting users to explore alternatives. This trend is reinforced by Regulation (EU) 2017/852 [7] which mandates the phase-out of certain mercury containing products, including HPS lights, within the European Union, unless specific exemption criteria are met.

LEDs used in horticulture are optimized to enhance plant growth and quality while minimizing energy consumption by employing targeted wavelengths, particularly (far-)red and/or blue light. These wavelengths, along with light intensity, have been shown to influence the virulence of various plant pathogens, including bacteria, fungi and viruses [8–16]. However, in plant pathology and diagnostics, the primary objective is to study, detect or identify pathogens. Consequently, the lighting conditions must be tailored to support pathogen virulence and enhance the visibility of disease symptoms rather than promoting plant growth. For virus detection, bioassays involve inoculating susceptible test plants with sap from infected plant material. Bacterial identification typically involves a pathogenicity test, where plants are inoculated with cells from a pure bacterial culture suspected to be the etiological agent of the disease [17]. To simplify terminology, the term disease assay is used here to encompass both virus detection bioassays and bacterial pathogenicity tests. Fine-tuning of the light spectrum and intensity may enhance the expression of pathogen symptoms, thereby increasing the accuracy and effectiveness of plant pathogen diagnostics.

This study aimed to identify suitable LEDs for plant virus and bacteria disease assays, facilitating the transition from HPS to LED technology in plant pathogen diagnostics. Two types of LEDs were evaluated to assess the impact of different light spectra on assay outcomes. A variety of test plants, viruses and bacteria were included to capture a wide range of symptoms. The comparison demonstrated that “broad spectrum with far-red” LEDs could serve as a viable alternative to HPS lights for disease assays of plant viruses and bacteria.

## Materials and methods

### Greenhouse set-up

Two comparable test compartments were used for comparison studies, one equipped with LEDs and the other with HPS lights. The sides were covered with opaque white plastic to prevent light transmission between compartments. Experiments were conducted during the winter season to minimize the influence of natural daylight. Supplemental lighting was programmed to maintain a 14-hour photoperiod. Greenhouse settings included a night-day temperature of 18–25 °C and a relative humidity (RH) of 70%, except for *Ralstonia* spp. assays with a night-day temperature of 26–30°C and >80% RH.

### Light specifications

HPS lights (Philips Master SON-T PIA Plus, 400 W) were compared with two types of LEDs: “daylight spectrum” LEDs (HORTILED top 120v19, 336 W, dimmable), and “broad spectrum with far-red” LEDs (Fluence VYPR 3p R6 + FR, 600 W, dimmable). After the first experiment (B1), the LEDs were dimmed to match the levels of the HPS lights. Light intensity was measured at multiple points within each compartment at plant level using a LI-COR Quantum/Radiometer/ Photometer model LI-189 in the absence of daylight. Spatial variations in light intensity were observed with ranges indicated in Tables 1 and 2, with lower intensities particularly at the compartment edges. To control for the potential effect of these spatial variations, plant-pathogen combinations were placed in similar positions across both test compartments. The light spectra for each system were measured without daylight, using an Asensetek ALP-01 spectrometer. Compartments were equipped with 8 LED or 10 HPS fixtures ensuring comparable light homogeneity.

**Table 1:** Greenhouse lighting conditions, and virus-plant combinations per experiment.

		Experiment V1a	Experiment V1b	Experiment V2
	LED spectrum	Daylight	Daylight	Broad with far-red
	Approximate light intensity (µmol/m^2^/s)	LED: 60–78 HPS: 45–60	LED: 52–61 HPS: 45–63	LED: 36–64 HPS: 43–70
	Symptom observation period	Week 49 2021-week 4 2022	Week 8–12 2022	Week 48 2022-week 2 2023
Virus^1^	Isolate	Test plants^2^		
TRSV	9702383	*C. quinoa* *C. sativus* *N. benthamiana* *N. occidentalis* *N. tabacum*	*N. benthamiana* *N. occidentalis* *N. tabacum*	*C. quinoa* *C. sativus* *N. benthamiana* *N. occidentalis* *N. tabacum*
TSWV	21007721	*D. stramonium* *N. benthamiana* *N. glutinosa* *N. occidentalis* *N. tabacum*	*–*	*D. stramonium* *N. benthamiana* *N. glutinosa* *N. occidentalis* *N. tabacum*
PhCMoV	33226137	*N. benthamiana* *N. occidentalis*	*N. benthamiana* *N. occidentalis*	*N. benthamiana* *N. occidentalis*

^1^TRSV: tobacco ringspot virus (*Nepovirus nicotianae*), TSWV: tomato spotted wilt virus (*Orthotospovirus tomatomaculae*), PhCMoV: Physostegia chlorotic mottle virus (*Alphanucleorhabdovirus physostegiae*).

^2^Full names: *Chenopodium quinoa, Cucumis sativus “*Chinese Slangen*”, Datura stramonium, Nicotiana benthamiana, Nicotiana glutinosa, Nicotiana occidentalis* “P1”, *Nicotiana tabacum “*White Burley”.

**Table 2:** Greenhouse lighting conditions, and bacteria-plant combinations per experiment.

		Experiment B1	Experiment B2	Experiment B3
	LED spectrum	Daylight	Daylight	Broad with far-red
	Approximate light intensity (µmol/m2/s)	LED: 75–131 HPS: 43–71	LED: 60–78 HPS: 45–60	LED: 36–64 HPS: 43–70
	Symptom observation period	Week 51 2020 – week 8 2021	Week 51 2021-week 6 2022	Week 49 2022-week 6 2023
Bacteria^1^	Isolate	Test plants^2^		
*R. solanacearum* ph II (10)	PD2762 PD7465^3^ PD7466^3^	*S. lycopersicum* (1)	*S. lycopersicum* (2)	*S. lycopersicum* (2)
*R. pseudosolanacearum* ph I (10)	PD7123	*S. lycopersicum* (1)	*S. lycopersicum* (2)	*S. lycopersicum* (2)
*C. sepedonicus* (10)	PD406 PD7861	*S. melongena* (2)	*S. melongena* (2)	*S. melongena* (2)
*X. citri* pv. *aurantifolii* (2) *X. citri* pv. *citri* (2)	PD6315 PD990	–	–	*C. sinensis* (1) *C. calamondin* (1)
*P. syringae* pv. *syringae* (2)	PD 760	–	*N. tabacum* (1)	*N. tabacum* (1)

^1^Full names: *Clavibacter sepedonicus*, *Pseudomonas syringae* pv. *syringae*, *Ralstonia pseudosolanacearum* phylotype I, *Ralstonia solanacearum* phylotype II, *Xanthomonas citri* pv. *aurantifolii*, *Xanthomonas citri* pv. *citri*; number of biological replicates (inoculated/infiltrated plants/leaves) indicated in brackets.

^2^Full names: *Citrus calamondin* “Oriana”, *Citrus sinensis* “Aurancio”, *Nicotiana tabacum* “White Burley”, *Solanum lycopersium* “Money Maker”, *Solanum melongena* “Black Beauty”; number of technical replicates indicated in brackets.

^3^Isolates PD7465 and PD7466 were not included in experiment B3.

### Plant cultivation

All plants were grown in fertilized compost soil. For virus bioassays, seeds of various test plant species (Table 1) were initially sown in a greenhouse compartment under HPS lights. After two weeks, the seedlings were transplanted into 11 cm diameter pots within the respective test compartments, while *Cucumis sativus* seeds were sown directly into the 11 cm pots. For bacterial pathogenicity tests, seeds were sown directly in the test compartments (Table 2). After two weeks, *Solanum lycopersicum* and *S. melongena* were transplanted into trays (10 plants/tray) and *Nicotiana tabacum* were transplanted into 11 cm pots. Mature citrus plants (*C. sinensis* and *C. calamondin*) that had been growing for approximately 18 months under HPS lights at a night-day temperature of 18–22 °C and 70% RH were pruned. After pruning, the citrus plants were transferred to the test compartments to allow the development of young, fully-expanded leaves for a detached leaf assay.

### Virus detection bioassays

Virus–plant combinations for each experiment are detailed in Table 1. For each virus, four plants per species per compartment were mechanically inoculated [18] with the same inoculum. Additionally, four plants per species per compartment were inoculated with buffer only. For *C. sativus,* cotyledons of eight plants were inoculated. All plants were assessed for local and systemic disease symptoms twice a week, until no further symptom progression was observed, with a minimum period of three weeks. In cases where symptoms of Physostegia chlorotic mottle virus (PhCMoV) were unclear, DAS-ELISA [19] was performed on young, uninoculated leaf samples from individual test plants using a polyclonal antiserum specific to PhCMoV (kindly provided by Julius Kühn-Institute, Germany). Negative controls included pooled samples from buffer-inoculated plants of the same species.

### Bacterial pathogenicity tests

Bacteria-plant combinations for each experiment are detailed in Table 2. Bacterial isolates (Table 2) were grown on Nutrient Agar (NA), Yeast Extract–Peptone–Glycerol (YPG) or Wilbrink medium under standard laboratory conditions. Depending on the growth speed of the isolates, two-to-five-day old pure cultures were suspended in 0.01 M phosphate buffer (PB). Multiple isolates of some bacterial species were included, because of a high variation in virulence. Symptoms were evaluated at the end of each experiment. The pathogenicity of bacterial isolates on each host species was confirmed in advance. For *Ralstonia* and *Clavibacter,* ten plants per isolate were inoculated per compartment [20]. For *Xanthomonas,* young, fully-expanded leaves were used in a detached leaf assay [21]. For *Pseudomonas*, one leaf of a plant at the stage of two true leaves was infiltrated with a bacterial suspension under laboratory conditions, as previously described for *R. pseudosolanacearum* [22]. For each bacteria-plant combination (Table 2, Experiment B1), re-isolation was performed by selecting one symptomatic plant [23]. For re-isolation of *C. sepedonicus*, YPG plates were incubated at 21^o^C until colonies with a typical morphology of the initially inoculated isolate appeared. These colonies were selected and tested with real-time PCR [24] to confirm their identity.

## Results

**Fig 1:**
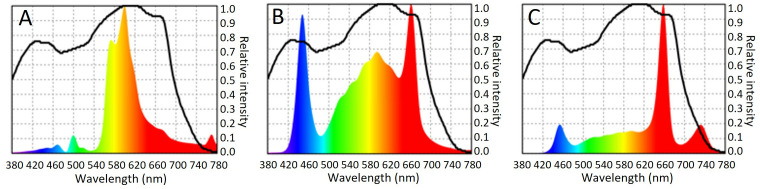
Spectral measurement of light composition (photosynthetic flux density) showing the relative intensity of each wavelength for A) HPS lights (Philips Master SON-T PIA Plus), B) “Daylight spectrum” LEDs (HORTILED top 120v19), C) “Broad spectrum with far-red” LEDs (Fluence VYPR 3p R6 + FR). Black line: McCree action spectrum, indicating the wavelength composition most effectively utilized by plants for photosynthesis.

The light spectra of the HPS lights and LEDs compared in this study are shown in Fig 1. The HPS lights predominantly emit yellow and orange light, accompanied by minor peaks of blue, green, and infrared (Fig 1A). In contrast, the “daylight spectrum” LEDs exhibit peaks in both blue and red wavelengths (Fig 1B), whereas the “broad spectrum with far-red“LEDs emit a substantial red peak, with minor peaks in blue and infrared (Fig 1C).

The “daylight spectrum” LEDs caused notable deviations in symptom development compared to HPS lights, particularly in virus-infected plants. In bacterial assays, the most pronounced symptom differences occurred when the LEDs were operated at a relatively high light intensity. These issues were resolved with the “broad spectrum with far-red” LEDs at a light intensity similar to HPS, resulting in comparable symptoms to those observed under HPS lights (Table 3).

**Table 3:** Effects of the tested LEDs on the symptomatology of test plants, compared to HPS lights, following inoculation with viruses and bacteria.

Type of LED	Daylight			Broad with far-red
**Light intensity (µmol/m2/s)**	**75-131**	**60-78**	**52-61**	**36-64**
**Viruses**	Not tested	Most symptoms less severe, delayed and/or on less plants (experiment V1a)	Most symptoms less severe, delayed and/or on less plants (experiment V1b)	Comparable symptoms (experiment V2)
**Bacteria**	Most symptoms less severe, delayed, and/or on less plants (experiment B1)	Symptoms slightly less severe, slightly delayed, and/or on less plants (experiment B2)	Not tested	Comparable symptoms (experiment B3)

### Daylight spectrum

Virus disease assays performed under “daylight spectrum” LEDs (experiment V1a) resulted in delayed and less pronounced virus symptoms on fewer test plants compared to HPS lights. For TRSV (S1A Table), notable differences in symptom development were observed: on *Nicotiana* spp., the typical concentric rings associated with nepovirus infections were less distinct under LEDs compared to HPS lights. Additionally, symptoms on *Chenopodium quinoa*, including necrotic spots and top necrosis, were less severe under LEDs. In contrast, symptoms on *C. sativus*, such as chlorotic lesions, mosaic patterns and growth reduction, were more pronounced under LEDs.

Regarding TSWV (S1B Table), various symptoms on *N. benthamiana*, *N. tabacum*, *Datura stramonium*, and *N. glutinosa* appeared a few days later, were less distinct, and/or visible on slightly fewer plants when exposed to LEDs as compared to HPS lights. On *N. occidentalis*, the disparities between the two lighting types were minimal.

The largest differences in symptom development were observed for PhCMoV (S1C Table). Under LEDs, only one *N. benthamiana* plant showed symptoms, including rugosity and growth reduction, whereas under HPS lights clear symptoms appeared on all four plants and at earlier time points. The remaining three plants under LEDs exhibited only faint symptoms. PhCMoV was only detected in three out of four plants under LEDs by DAS-ELISA (S2 Table). For *N. occidentalis*, only two plants displayed chlorotic veins under LEDs, whereas under HPS distinct chlorotic veins and growth inhibition were observed on all four plants at an earlier timepoint. PhCMoV was only detected in the two symptomatic plants under LEDs by DAS-ELISA (S2 Table). All four *N. occidentalis* plants under LEDs also showed chlorosis on the leaves of the flowering stem. However, since this type of chlorosis was also observed on the four control plants inoculated with buffer only, these symptoms were not virus-induced as confirmed by DAS-ELISA. In conclusion, PhCMoV-induced symptoms under LEDs were not always clear, though based on DAS-ELISA three out of four *N. benthamiana* and two out of four *N. occidentalis* plants were infected. In contrast, under HPS, all plants became infected based on their clear symptoms.

To exclude potential effects of light intensity, LEDs were dimmed to 16% of their maximum output to better match the intensity of the HPS lights (experiment V1b). This adjustment was made because the LED light intensity in experiment V1a was approximately 35% higher than that of the HPS lights. A partial repetition of experiment V1a was conducted, focusing on the inoculations that showed large differences between LEDs and HPS (Table 1). However, due to longer daylight hours and an increase in sunny days compared to experiment V1a, the lights were activated for a shorter duration. Nonetheless, the (concentric) rings indicave of TRSV infection remained less pronouned or appeared later under LEDs compared to HPS on *N. benthamiana* and *N. tabacum*. No such differences were observed for *N. occidentalis*. For PhCMoV on *N. benthamiana*, symptoms appeared slightly later and no growth inhibition was observed under LEDs. On *N. occidentalis*, vein chlorosis was visible on only one out of four plants under LEDs, compared to three out of four plants under HPS lights (S3 Table). Clearly, differences in virus symptomatology between LEDs and HPS lights persisted, even with more similar light intensities.

In bacterial disease assays under “daylight spectrum” LEDs (experiments B1 and B2), symptom development was (slightly) delayed, symptoms were (slightly) less severe, and fewer plants were affected compared to HPS lights (S6 and S7 Tables).

Under undimmed LEDs (experiment B1), *R. solanacearum* phylotype II isolate PD2762 induced typical wilting symptoms on a lower number of *S. lycopersicum* plants compared to HPS lights (S6B Table). Several plants remained symptom-free, or symptoms were unclear (S6B Table). However, isolates PD7465 and PD7466 and *R. pseudosolanacearum* phylotype I isolate PD7123, induced wilting symptoms in all plants under undimmed daylight spectrum LEDs and HPS lights (S6B Table). Under dimmed daylight spectrum LEDs (experiment B2) all *R. solanacearum* or *R. pseudosolanacearum* isolates induced typical wilting symptoms on all plants (S7B Table). Although not quantified, *S. lycopersicum* plants showed delayed symptom development and less severe symptoms under daylight spectrum LEDs than HPS, irrespective of the isolate. However, this delayed symptom development was less pronounced in experiment B2 (dimmed daylight spectrum LED) than in experiment B1 (undimmed daylight spectrum LED). Both *Ralstonia* pathogens were confirmed as the causal agent of the wilting symptoms in experiment B1.

For *C. sepedonicus,* a slightly lower number of *S. melongena* plants showed disease symptoms under undimmed LEDs (experiment B1) compared to HPS lights (S6A Table). Under the dimmed LEDs (experiment B2), isolate PD7861 induced symptoms in a comparable number of plants by the end of the experiment as under HPS lights (S7A Table). However, for isolate PD406 a higher number of plants remained symptom-free (S7A Table). Symptom development was delayed under LEDs, especially when undimmed (experiment B1). Plants under LEDs took several additional days to develop the typical leaf senescence and collapse symptoms, but this was not quantified, as plants were only assessed at the end of the experiment when most plants developed clear symptoms. *C. sepedonicus* was confirmed as the causal agent of the induced symptoms (experiment B1). In addition, the severity of the symptoms was slightly reduced under LEDs compared to HPS.

Upon leaf infiltration with *P. syringae* pv. *syringae*, *N. tabacum* leaves showed a comparable strong hypersensitive response (HR) 24 h post-infiltration, irrespective of the light conditions under which the plants were previously grown (S7C Table).

Differences in plant growth and development were observed under LEDs compared to HPS, though these observations were not quantified. Some plants grown under LEDs appeared slightly larger and had a darker green color (experiment V1). Plant growth was highly promoted when plants were exposed to the undimmed LEDs (experiment B1) with light intensity nearly twice as high as that of the HPS lights (Table 2). This effect was particularly evident in *S. melongena*, which also showed pronounced yellowing. In general, plants grown under LEDs (experiment B1 and B2) developed firmer stems, a broader structure, larger leaves, and increased side-branching. These traits were less pronounced under dimmed LEDs (experiment B2).

### Broad with far-red spectrum

Virus disease assays under “broad spectrum with far-red” LEDs, which have a spectral composition more similar to that of HPS lights, resulted in symptoms comparable to those observed under HPS lights (S4 Table). For PhCMoV, several symptoms appeared earlier or were more prevalent under LEDs, in contrast to experiment V1 (“daylight spectrum”), where only some plants under LEDs showed (clear) symptoms. PhCMoV symptom development under HPS was consistent with the observations in experiment V1.

Bacterial disease assays under the “broad spectrum with far-red” LEDs also gave comparable results to those observed under HPS lights (experiment B3, S8 Table). Unlike the “daylight spectrum” LEDs (experiment B1 and B2), no delay of disease symptom development or severity of symptoms was observed, and the number of plants with symptoms was almost the same as under HPS lights (S8 Table).

*X. citri pv. citri* (S8C Table) induced typical symptoms, including glassy tissue and development of corky lesions at the site of infiltration, and pronounced leaf chlorosis often leading to necrosis, under both light conditions. Symptoms were slightly more pronounced on leaves grown under LEDs. For *X. citri pv. aurantifolii*, similar results were obtained in *C. calamondin “Oriana”*, while no symptoms were observed in *C. sinensis “Aurancio”* under either light condition. *N. tabacum* leaf infiltration with *P. syringae* pv. *syringae* induced a strong hypersensitive response, regardless of prior light conditions (S8D Table).

In contrast to the observations under “daylight spectrum” LEDs, plant growth and development of test plants, prior to inoculation, were highly similar between “broad spectrum with far-red” LEDs and HPS.

## Discussion

The results of these comparative studies between HPS lights and two types of LEDs revealed that not all LEDs are suitable for disease assays involving plant pathogenic viruses and bacteria. LED horticultural lighting is typically optimized for plant growth and quality, but spectral composition also influences plant secondary metabolism and defense mechanisms [25,26]. Unlike HPS lights, which primarily emit yellow and orange light with minor peaks in blue, green, and infrared (Fig 1A), the “daylight spectrum” LEDs featured pronounced peaks in both blue and red wavelengths (Fig 1B). These wavelengths aligned more closely with the McCree action spectrum [27], representing the wavelengths most efficiently used in photosynthesis and likely contributed to increased plant size, although size differences were not quantified. Red light supplemented with blue light is associated with enhanced biomass production in rice [28], whereas the blue light peak may explain the yellow pigmentation observed in *S. melongena* leaves, as excessive chlorotic areas have been reported in tomato leaves under continuous monochromatic blue light [29]. Furthermore, blue light has been shown to increase carotenoid content in baby leaf lettuce [30] and Chinese cabbage [31], which supports photosynthesis and protects the photosystem from reactive oxygen species damage [32].

In addition to the spectrum, light intensity was also found to influence plant size. At full intensity, the “daylight spectrum” LEDs enhanced the growth of *S. lycopersicum* and *S. melongena* compared to dimmed LEDs. In *S. melongena*, elevated light intensity has been associated with an increased photosynthetic response [16]. Similarly, fresh leaf weight of red lettuce increased by 78% when LED light intensity rose from 130 µmol m^−2^ s^−1^ to 389 µmol m^−2^ s^−1^, due to increased photosynthesis [33]. In contrast to the “daylight spectrum” LEDs, “broad spectrum with far-red” LEDs feature a red-light peak and an additional far-red peak but lack a dominant blue light peak (Fig 1C), and resulted in similar plant development and growth as under HPS lighting.

Since the primary objective of this study was to identify LEDs under which disease symptoms are clearly expressed, the symptoms of plants inoculated with various viruses and bacteria were compared with those obtained under HPS lights. Under “daylight spectrum” LEDs, virus symptoms were generally less pronounced or even absent compared to those observed under HPS lights for various virus-plant combinations (experiment V1). For example, approximately half of the plants inoculated with PhCMoV showed no signs of infection under daylight spectrum LEDs, whereas all plants inoculated under HPS lights displayed clear symptoms. Similar challenges with virus symptom expression under LED lighting were reported in a different laboratory [34]. Regarding bacteria, symptoms developed on most of the inoculated test plants under “daylight spectrum” LEDs, but symptom onset was delayed, and severity was reduced, especially under high light intensities (experiments B1 and B2).

To assess whether light intensity affected virus symptom development, experiment V1b was conducted using a light intensity more closely matching that of HPS lights. Due to extended daylight hours, experiment V1b (February-March) required fewer hours of supplemental lighting compared to experiments V1a, B1 and B2 (December-January). Despite this difference, disparities in symptom development persisted between LEDs and HPS in experiment V1b, similar to those observed in experiment V1a. Fewer plants showed symptoms, or symptoms were less pronounced, and appeared later under the “daylight spectrum” LEDs compared to HPS. The persistent differences between LEDs and HPS indicates that the higher LED light intensity in experiment V1a did not account for the significant deviations in virus symptomatology between LED and HPS.

As spectral composition has been shown to influence pathogen resistance, further experiments were performed with “broad spectrum with far-red” LEDs, with a spectrum more comparable to that of HPS lights. For both viruses and bacteria, symptoms developed similarly to HPS lights (Experiment V2 and B3). For PhCMoV, symptom development typically takes longer than for other plant viruses and, therefore, plants were monitored for an extended period. Five to six weeks post-inoculation, chlorotic symptoms appeared not only on virus-inoculated plants but also on negative controls. This suggested a physiological origin of these symptoms, likely due to nutrient deficiencies. These physiological symptoms were more pronounced under LEDs than under HPS. Without negative controls, these physiological symptoms could have been mistaken for virus-related symptoms, underscoring the importance of including negative controls [35], especially in long-duration assays. The influence of spectral composition, especially the delay in symptom development with increased blue light, was also found by Schuerger and Brown [14], who reported that symptoms of tomato mosaic virus appeared later and were less severe under light with blue and UV-A wavelengths, compared to light sources lacking these wavelengths. Also, cucumber mosaic virus symptoms were less pronounced under red and blue light, than under white light [10], which resembles the decrease in symptom severity observed in this study under the “daylight spectrum” LEDs with red and blue light peaks. In contrast, supplemental far-red light increased disease severity of *B. cinerea*, *Phytophthora infestans* and *Pseudomonas syringae* in tomato [36]. Similarly, in this study, enhanced symptom development was observed when far-red light was incorporated into the light spectrum. However, spectral composition has been shown to affect different pathosystems in varying ways [14,16].

Regarding energy consumption, the “broad spectrum with far-red” LEDs had a maximum power output of 600 W compared to 400 W for the HPS lights. By operating the LEDs at 16% of their maximum power to match the light intensity of HPS, energy consumption was reduced by approximately 75% (0.16 * 600 W for LED vs. 400 W for HPS). While reduced heat output from the LEDs in winter may slightly increase heating costs, the overall energy use in a greenhouse is expected to be lower with LEDs than HPS [2]. Additionally, the perceived temperature in LED-lit compartments is lower than in those with HPS lights, due to the reduced radiant heat from LEDs, which may be more comfortable for greenhouse staff [37].

Concluding, light intensity was shown to influence bacterial symptoms, whereas spectral composition was the more critical factor for virus symptoms. For both bacteria and viruses, disease assays performed under dimmed “broad spectrum with far-red” LEDs, gave the best results, comparable to HPS lights. Furthermore, these LEDs facilitated visual assessment of disease symptoms because the light appears more white in comparison to the yellow/orange light emitted by HPS, due to its more balanced spectrum and the inclusion of green light [38]. Since the primary aim of this study was to identify LEDs that could replace HPS lights in disease assays, this study does not provide an exhaustive comparison of different lighting options. Nevertheless, it underscores the critical role of light intensity and spectrum in successful disease assays, which should be considered when transitioning to new types of light sources. Overall, the findings indicate that “broad spectrum with far-red” LEDs can serve as a suitable alternative to HPS lights for disease assays on plant pathogenic viruses and bacteria.

## Supporting information

S1 Table Experiment V1a: symptom development under HPS and “daylight spectrum” LEDs on test plants inoculated with (A) tobacco ringspot virus, (B) tomato spotted wilt virus, and (C) Physostegia chlorotic mottle virus. (XLSX)

S2 Table Experiment V1a: systemic symptoms and DAS-ELISA results of plants inoculated with Physostegia chlorotic mottle virus (isolate 33226137) under “daylight spectrum” LEDs. (XLSX)

S3 Table Experiment V1b: symptom development under HPS and “daylight spectrum” LEDs on test plants inoculated with (A) tobacco ringspot virus, and (B) Physostegia chlorotic mottle virus. (XLSX)

S4 Table Experiment V2: symptom development under HPS and “broad spectrum + far-red” LEDs on test plants inoculated with (A) tobacco ringspot virus, (B) tomato spotted wilt virus, and (C) Physostegia chlorotic mottle virus. (XLSX)

S5 Table Experiment V1 and V2: symptom abbreviations. (XLSX)

S6 Table Experiment B1: symptom evaluation under HPS and undimmed “daylight spectrum” LEDs on test plants inoculated with (A) *Clavibacter sepedonicus*, and (B) *Ralstonia pseudosolanacearum* phylotype I and *Ralstonia solanacearum* phylotype II. (XLSX)

S7 Table Experiment B2: symptom evaluation under HPS and dimmed “daylight spectrum” LEDs on test plants inoculated with (A) *Clavibacter sepedonicus*, (B) *Ralstonia pseudosolanacearum* phylotype I and *Ralstonia solanacearum* phylotype II and (C) *Pseudomonas syringae* pv. *syringae.* (XLSX)

S8 Table Experiment B3: symptom evaluation under HPS and “broad spectrum with far-red” LEDs on test plants inoculated with (A) *Clavibacter sepedonicus*, (B) *Ralstonia pseudosolanacearum* phylotype I and *Ralstonia solanacearum* phylotype II, (C) *Xanthomonas citri* pv. *aurantifolii* and pv. *citri* and (D) *Pseudomonas syringae* pv. *syringae.* (XLSX)
