# Supplementary material for: Comparison and selection of light-emitting diodes (LEDs) for disease assays on plant pathogenic viruses and bacteria in greenhouses
Source: PLoS One. 2025 Aug 11;20(8):e0328277. doi: 10.1371/journal.pone.0328277 (PMC12338770; doi:10.1371/journal.pone.0328277)
Supplement: Attachment — Submitted filename: Reviewer Comment 1.docx [file pone.0328277.s009.docx]

Reviewer Comments

**Report**

The manuscript “**Comparison and selection of light-emitting diodes (LEDs) for disease assays on plant pathogenic viruses and bacteria in greenhouses**” is structured and written well. There are numerous strengths in the study, including its novel technique to analyze the disease on plant pathogenic viruses and bacteria using different diodes experiments that have been carried out. Overall, I consider this to be a good quality manuscript but an addition of few details and clarification can make it an excellent contribution to the mitigation techniques. Specific comments are given below.

Specific Comments

Comment 1: in introduction section, author mentioned diseases. Author should provide few lines with the detail of diseases and their causative agents for better understanding

Comment 2: Table no 1- I suggested that Author can remove the blank column and row that could be a neat presentation and avoid the mix-ups.

Comment 3: Discussion – author kindly describe the nature of plant varieties which subjected to analysis.

Comment 4: Author should compare the previous report for enhancing the current research values and strong scientific supports.

Comment 5: what is the significant result obtained through the analysis.

Comment 6: In Results and discussion, author should check the sentence as well.

Comment 7: There are many grammatical mistakes that need to be rectified before submitting for publication.

Comment 8: The authors must check if they have given the citations, references, Figure legends and table legends according to the format given in the Journal guide lines.
