# Supplementary material for: Comparison and selection of light-emitting diodes (LEDs) for disease assays on plant pathogenic viruses and bacteria in greenhouses
Source: PLoS One. 2025 Aug 11;20(8):e0328277. doi: 10.1371/journal.pone.0328277 (PMC12338770; doi:10.1371/journal.pone.0328277)
Supplement: Attachment — Submitted filename: 20250514_Response to reviewers.docx [file pone.0328277.s011.docx]

Dear editor, dear reviewers,

Thank you for the time and effort you have dedicated to evaluating our manuscript. We appreciate the feedback and have worked carefully to revise the manuscript in response to the suggestions provided.

While we have addressed all comments to the best of our ability, we are concerned that some of the feedback may reflect a limited familiarity with the specific context of plant pathology and diagnostics. As a result, a few comments appear to be general or not fully aligned with the core focus of the manuscript. Nevertheless, we have revised the manuscript accordingly where appropriate.

Below, we provide a detailed response to each point raised by the reviewers (in **bold**).

Please note that I will be out of the office from May 28 until June 23, with limited access to my email.

Yours sincerely,

Anne Giesbers

Reviewer #1: Specific comments on the manuscript:
-The research results are superficial, lack novelty, and primarily consist of enumerative experiments.

**Authors: We are very surprised by this comment. This study comprised several years of novel work. As far as we know, there is no literature available on the effect of LED light versus HPS light on plant pathogen diagnostics.**

-The study’s objectives and scientific rationale are unclear.

**Authors: the study’s objectives and rationale are stated in the last paragraph of the Introduction, we have slightly rephrased this paragraph.**

-The tables and figures presented are difficult to interpret.

**Authors: We have adjusted the tables to improve consistency and readability.**

-The authors should invest more time in revising the manuscript. Key improvements should include:
• Clearly defining research goals.

**Authors: Research goals are defined in the last paragraph of the introduction.**

• Detailing the experimental methodology more thoroughly.

**Authors: We think that our Materials & Methods section is complete. Please elaborate which details you are missing if you do not agree.**

• Presenting results in a logical, scientifically grounded manner.

**Authors: We have improved some sentences for improved readability and consistency. We think our results are presented in a logical and scientifically grounded manner. Please elaborate and provide examples if you do not agree.**

• Condensing the Introduction and Conclusion to highlight key findings more effectively.

**Authors: We have rephrased several sentences to enhance clarity and better convey our message. The key findings are summarized in the final paragraph of the Discussion.**

Reviewer #2: The manuscript “Comparison and selection of light-emitting diodes (LEDs) for disease assays on plant pathogenic viruses and bacteria in greenhouses” is structured and written well. There are numerous strengths in the study, including its novel technique to analyze the disease on plant pathogenic viruses and bacteria using different diodes experiments that have been carried out. Overall, I consider this to be a good quality manuscript but an addition of few details and clarification can make it an excellent contribution to the mitigation techniques. Specific comments are given below.

**Authors: Thank you for appreciating our work and for providing feedback. We have done our best to improve the manuscript where needed.**

Specific Comments

Comment 1: in introduction section, author mentioned diseases. Author should provide few lines with the detail of diseases and their causative agents for better understanding

**Authors: A variety of bacteria and viruses were included in this study to capture a wide range of disease symptoms. A sentence on this has been added to the Introduction. We believe it is not relevant to further elaborate on the diseases that they cause, as this would distract from the primary goal of our study (the identification of suitable LEDs for disease assays).**

Comment 2: Table no 1- I suggested that Author can remove the blank column and row that could be a neat presentation and avoid the mix-ups.

**Authors: Thanks for the suggestion, this has been adjusted.**

Comment 3: Discussion – author kindly describe the nature of plant varieties which subjected to analysis.

**Authors: The plant varieties used in our study are well-established and widely utilized in plant virology and bacteriology research. As such, we feel that providing descriptions of these varieties would be redundant and would divert attention from the primary focus of the study.**

Comment 4: Author should compare the previous report for enhancing the current research values and strong scientific supports.

**Authors: We have incorporated references to relevant literature where applicable. However, as far as we know, there is no literature available on the effect of LED light versus HPS light on plant pathogen diagnostics.**

Comment 5: what is the significant result obtained through the analysis.

**Authors: the key findings are mentioned several times, for instance: Abstract (lines 26-28), Discussion (lines 331-342).**

Comment 6: In Results and discussion, author should check the sentence as well.
Comment 7: There are many grammatical mistakes that need to be rectified before submitting for publication.

**Authors: We have thoroughly checked our manuscript and improved wording throughout.**

Comment 8: The authors must check if they have given the citations, references, Figure legends and table legends according to the format given in the Journal guide lines.

**Authors: This has been checked and adjusted where needed.**

Reviewer #3: The research article titled "Comparison and selection of light-emitting diodes (LEDs) for disease assays on plant pathogenic viruses and bacteria in greenhouses " is an excellent contribution, and I hope it will attract the reader for future research. The authors have successfully evaluated LEDs for disease assays on plant pathogenic viruses and bacteria. But the manuscript has some major mistakes that the author needs to correct before the manuscript goes for publication. Some of the suggestions/comments are provided below to further improve the structure of the manuscript. I recommend it for publication (major revision) after carefully addressing the suggested comments.

**Authors: Thank you very much for recognizing the value of our work and for your feedback.**

1. Author should be adding keywords

**Authors: Keywords had already been provided: Bioassay; pathogenicity test; light intensity; light spectrum; high-pressure sodium (HPS) light; light-emitting diode (LED); test plants**

2. The abstract should be modified with your result. So, the author should be restructuring the abstract.

**Authors: the last sentence of the abstract mentions our main findings.**

3. In the Introduction, should be add the novelty and problem of the study

**Authors: The novelty and aim of the study are described in the last two paragraphs of the introduction.**

4. They should be discussed with recent literature

**We have incorporated references to relevant literature where applicable. However, as far as we know, there is no literature available on the effect of LED light versus HPS light on plant pathogen diagnostics.**

5. Should be add conclusion of the study

**Authors: The final paragraph of the Discussion serves as the conclusion of our study. We chose not to include a separate conclusion section, as we felt that integrating the concluding remarks within the Discussion allows for a more cohesive and fluid presentation of the findings.**

6. Should be include statistical analysis

**Authors: We believe that statistical analysis is not applicable to our study due to the nature of the data (qualitative observations) and the objectives of our research. Statistical testing would not provide any additional meaningful insights.**

7. The author should correct all the typo errors throughout the manuscript
8. author needs to improve the English corrections; most of the sentences are improper and hard to read.

**Authors: We have thoroughly checked our manuscript and improved wording throughout.**
