# Supplementary material for: Comparison and selection of light-emitting diodes (LEDs) for disease assays on plant pathogenic viruses and bacteria in greenhouses
Source: PLoS One. 2025 Aug 11;20(8):e0328277. doi: 10.1371/journal.pone.0328277 (PMC12338770; doi:10.1371/journal.pone.0328277)
Supplement: Attachment — Submitted filename: 20250625_Response to reviewers.docx [file pone.0328277.s012.docx]

Dear editor, dear reviewers,

Thank you for the time and effort you have dedicated to evaluating our manuscript. We have addressed the remaining comments below (in **bold**).

Yours sincerely,

Anne Giesbers

Reviewer #1: Comments to the authors:
- I have not really noticed the improvements in the article in the revised manuscript.
- The Results and Discussion section should clarify the impact of light on plant diseases.

**Authors: A variety of bacteria and viruses were included in this study to capture a wide range of disease symptoms. We believe it is not relevant to further elaborate on the diseases that these pathogens cause, as this would distract from the primary goal of our study: the identification of suitable LEDs for disease assays and diagnostics. Therefore, the focus is on symptom development.**

The author should logically rearrange the experiments, and the comparison results should be clearly stated instead of requiring the reader to search for information in the tables in the Supporting Information. The main results should be included in the results and discussion section of the paper, instead of in the supporting Information.

**Authors: The main results are summarized in Table 3 and discussed throughout the paper. Due to the number of individual experiments, we chose to include the data as a supplemental file.**

- Table S5 clearly shows the abbreviations. However, in tables S1-V1a, S2_V1a, S3_V1b, S4_V2, parameters such as dpi, index (4/4; 3/4...) are not defined or indicated in the experimental section, making it difficult to follow.

**Authors: Everything is explained in the table captions: “dpi: days post inoculation, number of test plants showing specific symptoms over the total number of inoculated plants indicated in brackets.”**

- The paper needs to clearly supplement the conclusion section.

**Authors: The final paragraph of the Discussion serves as the conclusion of our study. We chose not to include a separate conclusion section, as we felt that integrating the concluding remarks within the Discussion allows for a more cohesive and fluid presentation of the findings.**
