# Supplementary material for: Comparison and selection of light-emitting diodes (LEDs) for disease assays on plant pathogenic viruses and bacteria in greenhouses
Source: PLoS One. 2025 Aug 11;20(8):e0328277. doi: 10.1371/journal.pone.0328277 (PMC12338770; doi:10.1371/journal.pone.0328277)
Supplement: Peer review history

# Peer review: Decision Letter 0

**DOI:** 10.1371/journal.pone.0328277.r001

**Authors:** Karthik Kannan

7 May 2025

Dear Dr. Giesbers,

Thank you for submitting your manuscript to PLOS ONE. After careful consideration, we feel that it has merit but does not fully meet PLOS ONE’s publication criteria as it currently stands. Therefore, we invite you to submit a revised version of the manuscript that addresses the points raised during the review process.

Please submit your revised manuscript by Jun 21 2025 11:59PM. If you will need more time than this to complete your revisions, please reply to this message or contact the journal office at plosone@plos.org . When you're ready to submit your revision, log on to https://www.editorialmanager.com/pone/ and select the 'Submissions Needing Revision' folder to locate your manuscript file.

- A rebuttal letter that responds to each point raised by the academic editor and reviewer(s). You should upload this letter as a separate file labeled 'Response to Reviewers'.
- A marked-up copy of your manuscript that highlights changes made to the original version. You should upload this as a separate file labeled 'Revised Manuscript with Track Changes'.
- An unmarked version of your revised paper without tracked changes. You should upload this as a separate file labeled 'Manuscript'.

If you would like to make changes to your financial disclosure, please include your updated statement in your cover letter. Guidelines for resubmitting your figure files are available below the reviewer comments at the end of this letter.

If applicable, we recommend that you deposit your laboratory protocols in protocols.io to enhance the reproducibility of your results. Protocols.io assigns your protocol its own identifier (DOI) so that it can be cited independently in the future. For instructions see: https://journals.plos.org/plosone/s/submission-guidelines#loc-laboratory-protocols . Additionally, PLOS ONE offers an option for publishing peer-reviewed Lab Protocol articles, which describe protocols hosted on protocols.io. Read more information on sharing protocols at https://plos.org/protocols?utm_medium=editorial-email&utm_source=authorletters&utm_campaign=protocols .

We look forward to receiving your revised manuscript.

Kind regards,

Karthik Kannan, Ph. D.,

Academic Editor

PLOS ONE

Journal Requirements:

When submitting your revision, we need you to address these additional requirements.

1.Please ensure that your manuscript meets PLOS ONE's style requirements, including those for file naming. The PLOS ONE style templates can be found at

https://journals.plos.org/plosone/s/file?id=wjVg/PLOSOne_formatting_sample_main_body.pdf and

https://journals.plos.org/plosone/s/file?id=ba62/PLOSOne_formatting_sample_title_authors_affiliations.pdf

2. Please note that funding information should not appear in any section or other areas of your manuscript. We will only publish funding information present in the Funding Statement section of the online submission form. Please remove any funding-related text from the manuscript.

[Note: HTML markup is below. Please do not edit.]

Reviewers' comments:

Reviewer's Responses to Questions

**Comments to the Author**

1. Is the manuscript technically sound, and do the data support the conclusions?

Reviewer #1: No

Reviewer #2: Yes

Reviewer #3: Partly

**********

2. Has the statistical analysis been performed appropriately and rigorously?

Reviewer #1: No

Reviewer #2: Yes

Reviewer #3: No

**********

3. Have the authors made all data underlying the findings in their manuscript fully available?

The PLOS Data policy

Reviewer #1: Yes

Reviewer #2: Yes

Reviewer #3: Yes

**********

4. Is the manuscript presented in an intelligible fashion and written in standard English?

Reviewer #1: No

Reviewer #2: Yes

Reviewer #3: No

**********

Reviewer #1: Specific comments on the manuscript:

-The research results are superficial, lack novelty, and primarily consist of enumerative experiments.

-The study’s objectives and scientific rationale are unclear.

-The tables and figures presented are difficult to interpret.

-The authors should invest more time in revising the manuscript. Key improvements should include:

• Clearly defining research goals.

• Detailing the experimental methodology more thoroughly.

• Presenting results in a logical, scientifically grounded manner.

• Condensing the Introduction and Conclusion to highlight key findings more effectively.

Reviewer #2: The manuscript “Comparison and selection of light-emitting diodes (LEDs) for disease assays on plant pathogenic viruses and bacteria in greenhouses” is structured and written well. There are numerous strengths in the study, including its novel technique to analyze the disease on plant pathogenic viruses and bacteria using different diodes experiments that have been carried out. Overall, I consider this to be a good quality manuscript but an addition of few details and clarification can make it an excellent contribution to the mitigation techniques. Specific comments are given below.

Specific Comments

Comment 1: in introduction section, author mentioned diseases. Author should provide few lines with the detail of diseases and their causative agents for better understanding

Comment 2: Table no 1- I suggested that Author can remove the blank column and row that could be a neat presentation and avoid the mix-ups.

Comment 3: Discussion – author kindly describe the nature of plant varieties which subjected to analysis.

Comment 4: Author should compare the previous report for enhancing the current research values and strong scientific supports.

Comment 5: what is the significant result obtained through the analysis.

Comment 6: In Results and discussion, author should check the sentence as well.

Comment 7: There are many grammatical mistakes that need to be rectified before submitting for publication.

Comment 8: The authors must check if they have given the citations, references, Figure legends and table legends according to the format given in the Journal guide lines.

Reviewer #3: The research article titled "Comparison and selection of light-emitting diodes (LEDs) for disease assays on plant pathogenic viruses and bacteria in greenhouses " is an excellent contribution, and I hope it will attract the reader for future research. The authors have successfully evaluated LEDs for disease assays on plant pathogenic viruses and bacteria. But the manuscript has some major mistakes that the author needs to correct before the manuscript goes for publication. Some of the suggestions/comments are provided below to further improve the structure of the manuscript. I recommend it for publication (major revision) after carefully addressing the suggested comments.

1. Author should be adding keywords

2. The abstract should be modified with your result. So, the author should be restructuring the abstract.

3. In the Introduction, should be add the novelty and problem of the study

4. They should be discussed with recent literature

5. Should be add conclusion of the study

6. Should be include statistical analysis

7. The author should correct all the typo errors throughout the manuscript

8. author needs to improve the English corrections; most of the sentences are improper and hard to read.

**********

what does this mean? ). If published, this will include your full peer review and any attached files.

If you choose “no”, your identity will remain anonymous but your review may still be made public.

**Do you want your identity to be public for this peer review?** For information about this choice, including consent withdrawal, please see our Privacy Policy

Reviewer #1: No

Reviewer #2: **Yes: ** Dr.D.Swarna bharathi

Reviewer #3: **Yes: ** Dr. R. Krishnamoorthi,

Postdoctoral Researcher

Chang Gung University,

Taiwan

**********

[NOTE: If reviewer comments were submitted as an attachment file, they will be attached to this email and accessible via the submission site. Please log into your account, locate the manuscript record, and check for the action link "View Attachments". If this link does not appear, there are no attachment files.]

While revising your submission, please upload your figure files to the Preflight Analysis and Conversion Engine (PACE) digital diagnostic tool, https://pacev2.apexcovantage.com/ . PACE helps ensure that figures meet PLOS requirements. To use PACE, you must first register as a user. Registration is free. Then, login and navigate to the UPLOAD tab, where you will find detailed instructions on how to use the tool. If you encounter any issues or have any questions when using PACE, please email PLOS at figures@plos.org . Please note that Supporting Information files do not need this step.

Attachment Submitted filename: Reviewer Comment 1.docx

# Author comment: Author response to Decision Letter 1

**DOI:** 10.1371/journal.pone.0328277.r002

14 May 2025

Dear editor, dear reviewers,

Thank you for the time and effort you have dedicated to evaluating our manuscript. We appreciate the feedback and have worked carefully to revise the manuscript in response to the suggestions provided.

While we have addressed all comments to the best of our ability, we are concerned that some of the feedback may reflect a limited familiarity with the specific context of plant pathology and diagnostics. As a result, a few comments appear to be general or not fully aligned with the core focus of the manuscript. Nevertheless, we have revised the manuscript accordingly where appropriate.

Below, we provide a detailed response to each point raised by the reviewers.

Please note that I will be out of the office from May 28 until June 23, with limited access to my email.

Yours sincerely,

Anne Giesbers

Reviewer #1: Specific comments on the manuscript:

-The research results are superficial, lack novelty, and primarily consist of enumerative experiments.

Authors: We are very surprised by this comment. This study comprised several years of novel work. As far as we know, there is no literature available on the effect of LED light versus HPS light on plant pathogen diagnostics.

-The study’s objectives and scientific rationale are unclear.

Authors: the study’s objectives and rationale are stated in the last paragraph of the Introduction, we have slightly rephrased this paragraph.

-The tables and figures presented are difficult to interpret.

Authors: We have adjusted the tables to improve consistency and readability.

-The authors should invest more time in revising the manuscript. Key improvements should include:

• Clearly defining research goals.

Authors: Research goals are defined in the last paragraph of the introduction.

• Detailing the experimental methodology more thoroughly.

Authors: We think that our Materials & Methods section is complete. Please elaborate which details you are missing if you do not agree.

• Presenting results in a logical, scientifically grounded manner.

Authors: We have improved some sentences for improved readability and consistency. We think our results are presented in a logical and scientifically grounded manner. Please elaborate and provide examples if you do not agree.

• Condensing the Introduction and Conclusion to highlight key findings more effectively.

Authors: We have rephrased several sentences to enhance clarity and better convey our message. The key findings are summarized in the final paragraph of the Discussion.

Reviewer #2: The manuscript “Comparison and selection of light-emitting diodes (LEDs) for disease assays on plant pathogenic viruses and bacteria in greenhouses” is structured and written well. There are numerous strengths in the study, including its novel technique to analyze the disease on plant pathogenic viruses and bacteria using different diodes experiments that have been carried out. Overall, I consider this to be a good quality manuscript but an addition of few details and clarification can make it an excellent contribution to the mitigation techniques. Specific comments are given below.

Authors: Thank you for appreciating our work and for providing feedback. We have done our best to improve the manuscript where needed.

Specific Comments

Comment 1: in introduction section, author mentioned diseases. Author should provide few lines with the detail of diseases and their causative agents for better understanding

Authors: A variety of bacteria and viruses were included in this study to capture a wide range of disease symptoms. A sentence on this has been added to the Introduction. We believe it is not relevant to further elaborate on the diseases that they cause, as this would distract from the primary goal of our study (the identification of suitable LEDs for disease assays).

Comment 2: Table no 1- I suggested that Author can remove the blank column and row that could be a neat presentation and avoid the mix-ups.

Authors: Thanks for the suggestion, this has been adjusted.

Comment 3: Discussion – author kindly describe the nature of plant varieties which subjected to analysis.

Authors: The plant varieties used in our study are well-established and widely utilized in plant virology and bacteriology research. As such, we feel that providing descriptions of these varieties would be redundant and would divert attention from the primary focus of the study.

Comment 4: Author should compare the previous report for enhancing the current research values and strong scientific supports.

Authors: We have incorporated references to relevant literature where applicable. However, as far as we know, there is no literature available on the effect of LED light versus HPS light on plant pathogen diagnostics.

Comment 5: what is the significant result obtained through the analysis.

Authors: the key findings are mentioned several times, for instance: Abstract (lines 26-28), Discussion (lines 331-342).

Comment 6: In Results and discussion, author should check the sentence as well.

Comment 7: There are many grammatical mistakes that need to be rectified before submitting for publication.

Authors: We have thoroughly checked our manuscript and improved wording throughout.

Comment 8: The authors must check if they have given the citations, references, Figure legends and table legends according to the format given in the Journal guide lines.

Authors: This has been checked and adjusted where needed.

Reviewer #3: The research article titled "Comparison and selection of light-emitting diodes (LEDs) for disease assays on plant pathogenic viruses and bacteria in greenhouses " is an excellent contribution, and I hope it will attract the reader for future research. The authors have successfully evaluated LEDs for disease assays on plant pathogenic viruses and bacteria. But the manuscript has some major mistakes that the author needs to correct before the manuscript goes for publication. Some of the suggestions/comments are provided below to further improve the structure of the manuscript. I recommend it for publication (major revision) after carefully addressing the suggested comments.

Authors: Thank you very much for recognizing the value of our work and for your feedback.

1. Author should be adding keywords

Authors: Keywords had already been provided: Bioassay; pathogenicity test; light intensity; light spectrum; high-pressure sodium (HPS) light; light-emitting diode (LED); test plants

2. The abstract should be modified with your result. So, the author should be restructuring the abstract.

Authors: the last sentence of the abstract mentions our main findings.

3. In the Introduction, should be add the novelty and problem of the study

Authors: The novelty and aim of the study are described in the last two paragraphs of the introduction.

4. They should be discussed with recent literature

We have incorporated references to relevant literature where applicable. However, as far as we know, there is no literature available on the effect of LED light versus HPS light on plant pathogen diagnostics.

5. Should be add conclusion of the study

Authors: The final paragraph of the Discussion serves as the conclusion of our study. We chose not to include a separate conclusion section, as we felt that integrating the concluding remarks within the Discussion allows for a more cohesive and fluid presentation of the findings.

6. Should be include statistical analysis

Authors: We believe that statistical analysis is not applicable to our study due to the nature of the data (qualitative observations) and the objectives of our research. Statistical testing would not provide any additional meaningful insights.

7. The author should correct all the typo errors throughout the manuscript

8. author needs to improve the English corrections; most of the sentences are improper and hard to read.

Authors: We have thoroughly checked our manuscript and improved wording throughout.

Attachment Submitted filename: 20250514_Response to reviewers.docx

# Peer review: Decision Letter 1

**DOI:** 10.1371/journal.pone.0328277.r003

**Authors:** Karthik Kannan

9 Jun 2025

Dear Dr. Giesbers,

Thank you for submitting your manuscript to PLOS ONE. After careful consideration, we feel that it has merit but does not fully meet PLOS ONE’s publication criteria as it currently stands. Therefore, we invite you to submit a revised version of the manuscript that addresses the points raised during the review process.

Please submit your revised manuscript by Jul 24 2025 11:59PM. If you will need more time than this to complete your revisions, please reply to this message or contact the journal office at plosone@plos.org . When you're ready to submit your revision, log on to https://www.editorialmanager.com/pone/ and select the 'Submissions Needing Revision' folder to locate your manuscript file.

- A rebuttal letter that responds to each point raised by the academic editor and reviewer(s). You should upload this letter as a separate file labeled 'Response to Reviewers'.
- A marked-up copy of your manuscript that highlights changes made to the original version. You should upload this as a separate file labeled 'Revised Manuscript with Track Changes'.
- An unmarked version of your revised paper without tracked changes. You should upload this as a separate file labeled 'Manuscript'.

If you would like to make changes to your financial disclosure, please include your updated statement in your cover letter. Guidelines for resubmitting your figure files are available below the reviewer comments at the end of this letter.

If applicable, we recommend that you deposit your laboratory protocols in protocols.io to enhance the reproducibility of your results. Protocols.io assigns your protocol its own identifier (DOI) so that it can be cited independently in the future. For instructions see: https://journals.plos.org/plosone/s/submission-guidelines#loc-laboratory-protocols . Additionally, PLOS ONE offers an option for publishing peer-reviewed Lab Protocol articles, which describe protocols hosted on protocols.io. Read more information on sharing protocols at https://plos.org/protocols?utm_medium=editorial-email&utm_source=authorletters&utm_campaign=protocols .

We look forward to receiving your revised manuscript.

Kind regards,

Karthik Kannan, Ph. D.,

Academic Editor

PLOS ONE

[Note: HTML markup is below. Please do not edit.]

Reviewers' comments:

Reviewer's Responses to Questions

**Comments to the Author**

Reviewer #1: All comments have been addressed

Reviewer #2: All comments have been addressed

Reviewer #3: All comments have been addressed

**********

2. Is the manuscript technically sound, and do the data support the conclusions?

Reviewer #1: Partly

Reviewer #2: Yes

Reviewer #3: Yes

**********

3. Has the statistical analysis been performed appropriately and rigorously?

Reviewer #1: Yes

Reviewer #2: Yes

Reviewer #3: No

**********

4. Have the authors made all data underlying the findings in their manuscript fully available?

The PLOS Data policy

Reviewer #1: Yes

Reviewer #2: Yes

Reviewer #3: Yes

**********

5. Is the manuscript presented in an intelligible fashion and written in standard English?

Reviewer #1: Yes

Reviewer #2: Yes

Reviewer #3: Yes

**********

Reviewer #1: Comments to the authors:

- I have not really noticed the improvements in the article in the revised manuscript.

- The Results and Discussion section should clarify the impact of light on plant diseases. The author should logically rearrange the experiments, and the comparison results should be clearly stated instead of requiring the reader to search for information in the tables in the Supporting Information. The main results should be included in the results and discussion section of the paper, instead of in the supporting Information.

- Table S5 clearly shows the abbreviations. However, in tables S1-V1a, S2_V1a, S3_V1b, S4_V2, parameters such as dpi, index (4/4; 3/4...) are not defined or indicated in the experimental section, making it difficult to follow.

- The paper needs to clearly supplement the conclusion section.

Reviewer #2: Yes the author addressed the comments properly, which paved the way to recommend the article for acceptance.

Reviewer #3: The authors effectively addressed all the comments. I am satisfied with the author's response. Therefore, I may accept it for publication.

**********

what does this mean? ). If published, this will include your full peer review and any attached files.

If you choose “no”, your identity will remain anonymous but your review may still be made public.

**Do you want your identity to be public for this peer review?** For information about this choice, including consent withdrawal, please see our Privacy Policy

Reviewer #1: No

Reviewer #2: **Yes: ** Dr.D.Swarna bharathi

Reviewer #3: **Yes: ** Raman Krishnamoorthi

**********

[NOTE: If reviewer comments were submitted as an attachment file, they will be attached to this email and accessible via the submission site. Please log into your account, locate the manuscript record, and check for the action link "View Attachments". If this link does not appear, there are no attachment files.]

While revising your submission, please upload your figure files to the Preflight Analysis and Conversion Engine (PACE) digital diagnostic tool, https://pacev2.apexcovantage.com/ . PACE helps ensure that figures meet PLOS requirements. To use PACE, you must first register as a user. Registration is free. Then, login and navigate to the UPLOAD tab, where you will find detailed instructions on how to use the tool. If you encounter any issues or have any questions when using PACE, please email PLOS at figures@plos.org . Please note that Supporting Information files do not need this step.

# Author comment: Author response to Decision Letter 2

**DOI:** 10.1371/journal.pone.0328277.r004

25 Jun 2025

Reviewer #1: Comments to the authors:

- I have not really noticed the improvements in the article in the revised manuscript.

- The Results and Discussion section should clarify the impact of light on plant diseases.

Authors: A variety of bacteria and viruses were included in this study to capture a wide range of disease symptoms. We believe it is not relevant to further elaborate on the diseases that these pathogens cause, as this would distract from the primary goal of our study: the identification of suitable LEDs for disease assays and diagnostics. Therefore, the focus is on symptom development.

The author should logically rearrange the experiments, and the comparison results should be clearly stated instead of requiring the reader to search for information in the tables in the Supporting Information. The main results should be included in the results and discussion section of the paper, instead of in the supporting Information.

Authors: The main results are summarized in Table 3 and discussed throughout the paper. Due to the number of individual experiments, we chose to include the data as a supplemental file.

- Table S5 clearly shows the abbreviations. However, in tables S1-V1a, S2_V1a, S3_V1b, S4_V2, parameters such as dpi, index (4/4; 3/4...) are not defined or indicated in the experimental section, making it difficult to follow.

Authors: Everything is explained in the table captions: “dpi: days post inoculation, number of test plants showing specific symptoms over the total number of inoculated plants indicated in brackets.”

- The paper needs to clearly supplement the conclusion section.

Authors: The final paragraph of the Discussion serves as the conclusion of our study. We chose not to include a separate conclusion section, as we felt that integrating the concluding remarks within the Discussion allows for a more cohesive and fluid presentation of the findings.

Attachment Submitted filename: 20250625_Response to reviewers.docx

# Peer review: Decision Letter 2

**DOI:** 10.1371/journal.pone.0328277.r005

**Authors:** Karthik Kannan

30 Jun 2025

Comparison and selection of light-emitting diodes (LEDs) for disease assays on plant pathogenic viruses and bacteria in greenhouses

PONE-D-25-04310R2

Dear Dr. Giesbers,

We’re pleased to inform you that your manuscript has been judged scientifically suitable for publication and will be formally accepted for publication once it meets all outstanding technical requirements.

Within one week, you’ll receive an e-mail detailing the required amendments. When these have been addressed, you’ll receive a formal acceptance letter and your manuscript will be scheduled for publication.

An invoice will be generated when your article is formally accepted. Please note, if your institution has a publishing partnership with PLOS and your article meets the relevant criteria, all or part of your publication costs will be covered. Please make sure your user information is up-to-date by logging into Editorial Manager at Editorial Manager®  and clicking the ‘Update My Information' link at the top of the page. If you have any questions relating to publication charges, please contact our Author Billing department directly at authorbilling@plos.org.

If your institution or institutions have a press office, please notify them about your upcoming paper to help maximize its impact. If they’ll be preparing press materials, please inform our press team as soon as possible -- no later than 48 hours after receiving the formal acceptance. Your manuscript will remain under strict press embargo until 2 pm Eastern Time on the date of publication. For more information, please contact onepress@plos.org.

Kind regards,

Karthik Kannan, Ph. D.,

Academic Editor

PLOS ONE

Additional Editor Comments (optional):

Reviewers' comments:

Reviewer's Responses to Questions

**Comments to the Author**

Reviewer #2: All comments have been addressed

Reviewer #3: All comments have been addressed

**********

2. Is the manuscript technically sound, and do the data support the conclusions?

Reviewer #2: Yes

Reviewer #3: Yes

**********

3. Has the statistical analysis been performed appropriately and rigorously?

Reviewer #2: Yes

Reviewer #3: No

**********

4. Have the authors made all data underlying the findings in their manuscript fully available?

The PLOS Data policy

Reviewer #2: Yes

Reviewer #3: Yes

**********

5. Is the manuscript presented in an intelligible fashion and written in standard English?

Reviewer #2: Yes

Reviewer #3: Yes

**********

Reviewer #2: The review comments were properly addressed by the authors .

There is no additional queries needed to this extremely valuable manuscript.

I have accept the article for publication.

Reviewer #3: (No Response)

**********

what does this mean? ). If published, this will include your full peer review and any attached files.

If you choose “no”, your identity will remain anonymous but your review may still be made public.

**Do you want your identity to be public for this peer review?** For information about this choice, including consent withdrawal, please see our Privacy Policy

Reviewer #2: **Yes: ** Dr.D.Swarna bharathi

Reviewer #3: No

**********

# Editor report: Acceptance letter

**DOI:** 10.1371/journal.pone.0328277.r006

**Authors:** Karthik Kannan

PONE-D-25-04310R2

PLOS ONE

Dear Dr. Giesbers,

I'm pleased to inform you that your manuscript has been deemed suitable for publication in PLOS ONE. Congratulations! Your manuscript is now being handed over to our production team.

At this stage, our production department will prepare your paper for publication. This includes ensuring the following:

* All references, tables, and figures are properly cited

* All relevant supporting information is included in the manuscript submission,

* There are no issues that prevent the paper from being properly typeset

You will receive further instructions from the production team, including instructions on how to review your proof when it is ready. Please keep in mind that we are working through a large volume of accepted articles, so please give us a few days to review your paper and let you know the next and final steps.

Lastly, if your institution or institutions have a press office, please let them know about your upcoming paper now to help maximize its impact. If they'll be preparing press materials, please inform our press team within the next 48 hours. Your manuscript will remain under strict press embargo until 2 pm Eastern Time on the date of publication. For more information, please contact onepress@plos.org.

You will receive an invoice from PLOS for your publication fee after your manuscript has reached the completed accept phase. If you receive an email requesting payment before acceptance or for any other service, this may be a phishing scheme. Learn how to identify phishing emails and protect your accounts at https://explore.plos.org/phishing.

If we can help with anything else, please email us at customercare@plos.org.

Thank you for submitting your work to PLOS ONE and supporting open access.

Kind regards,

PLOS ONE Editorial Office Staff

on behalf of

Prof. Karthik Kannan

Academic Editor

PLOS ONE
